# Expression of Concern: LncRNA SNHG3 enhances the malignant progress of glioma through silencing KLF2 and p21

**DOI:** 10.1042/BSR-20180420_EOC

**Published:** 2020-07-30

**Keywords:** glioma, KLF2, p21, SNHG3

The Editorial Office has been made aware of potential issues surrounding the scientific validity of this paper, hence has issued an expression of concern to notify readers whilst the Editorial Office investigates.

